# Long-term fasting-induced parasympathetic and sympathetic autonomic nervous system modulation in a subgroup of the GENESIS study

**DOI:** 10.1038/s41366-025-01843-0

**Published:** 2025-07-09

**Authors:** Robin Mesnage, Alfred Holley, Franziska Grundler, Borja Martinez-Tellez, Françoise Wilhelmi de Toledo, Pierre Croisille

**Affiliations:** 1 Buchinger Wilhelmi Clinic, Überlingen, Germany; 2 Department of Nutritional Sciences, School of Life Course Sciences, Faculty of Life Sciences and Medicine, King’s College London, London, UK; 3 Department of Nursing, Physiotherapy and Medicine and SPORT Research Group (CTS-1024), CIBIS Research Center, University of Almería, Almería, Spain; 4 Department of Medicine, Division of Endocrinology and Einthoven Laboratory for Experimental Vascular Medicine, Leiden University Medical Center, Leiden, The Netherlands; 5 Université Lyon, UJM-Saint-Etienne, INSA, CNRS UMR 5520, INSERM U1206, CREATIS, Saint-Etienne, France; 6 Department of Radiology, University Hospital Saint-Etienne, Saint-Etienne, France

**Keywords:** Metabolism, Obesity

## Abstract

Parasympathetic and sympathetic nervous systems are known to be modulated during long-term fasting, but temporal dynamics and persistence post-fasting are unclear. We analyzed daily heart rate variability (HRV), as a reflection of autonomic nervous system balance, as an exploratory analysis, in 16 participants from the GENESIS trial following the Buchinger fasting protocol (12 days of ~250 kcal/day). HRV was assessed via Polar H10 sensors daily 7 days before, during and 7 days after fasting. Overall, fasting led to an increase in parasympathetic activity, as indicated by higher root mean square of successive differences (RMSSD) values, which rose from 27.16 ± 10.5 to 32.92 ± 17.65 ms (*p* < 0.001), alongside a reduction in sympathetic activity, with values decreasing from 0.39 ± 0.83 to 0 ± 1.05 (*p* < 0.001). Day-by-day analysis showed an initial rise in sympathetic activation during the early fasting phase (*p* < 0.05). Mental well-being improved significantly (*p* < 0.05), while sleep quality remained unchanged. These findings highlight fasting’s potential for autonomic regulation and stress resilience, with implications for clinical applications in stress-related disorders. Larger, randomized studies are needed to confirm these observations.

## Introduction

Heart rate variability (HRV) reflects the balance of the autonomic nervous system (ANS) which regulates sympathetic “fight or flight” and parasympathetic “rest and digest” activities. Long-term fasting triggers ANS shifts, though the temporality of these effects is not fully understood. Earlier work by Fahrner (1985) suggested that the reduction in sympathetic tone during prolonged fasting could underlie its beneficial effects on blood pressure regulation [1]. Studies reported a suppression of sympathetic tone in the first 24 h of fasting [2], followed by either a transient activation [3] or minimal impact [4] in the first 48 h of a fast, while an enhanced parasympathetic activity has been described during long-term fasting of more than 4 days [5]. The persistence of these adaptations during food reintroduction remains unclear.

We measured daily variations in HRV, 1 week before fasting, throughout the fasting period, and one week during the food reintroduction, as an exploratory study, in a subgroup of 16 participants from the single-arm interventional trial GENESIS.

## Methods

### Participants

The single-arm interventional trial GENESIS is already described in detail [6]. This fasting intervention was according to the Buchinger fasting protocol [7], a fluid-only modified fasting regimen [8], and consisted of 12 days during which the subjects received minimal caloric intake with ~250 kcal (daily fruit juice, vegetable soup, and honey) and undertook a physical activity programme including daily outdoor walks of 1.5 h and moderate-intensity fitness exercises. The study protocol was approved by the ethics board of the State Medical Chamber of Baden-Württemberg on 26.07.2021 and registered at ClinicalTrials.gov (NCT05031598). Informed consent was obtained from all subjects. This analysis includes a subset of 16 participants from the GENESIS trial who completed daily HRV recordings under standardized conditions throughout the study. No formal power calculation was performed, as this sub-analysis was exploratory in nature and limited to participants with complete and reliable HRV data across all study phases. Questionnaires included mental well-being documented by the Warwick-Edinburgh Mental Well Being Scale and quality of sleep by the Pittsburgh Sleep Quality Index.

### Heart rate variability analysis

ANS regulation was determined from morning heart rate variability recordings obtained in standardized conditions, with subjects asked to perform measurements always within minutes after waking up but while lying down, to avoid any confounding effects. Heart rate (HR) and RR intervals recordings of 4 min were achieved using Polar H10 sensor chest strap device (Polar Electro Oy, Kempele, Finland) paired with Kubios HRV mobile app, and HRV parameters were calculated using Kubios HRV Scientific software [9] (version 4.1.0, Kubios Oy, Kuopio, Finland). Root mean square of successive differences (RMSSD), is a time-domain parameter that reflects short-term heart rate variability and is mainly influenced by parasympathetic (vagal) activity, was derived from these recordings. High-frequency (HF) power is associated with parasympathetic modulation linked to respiratory sinus arrhythmia, whereas low-frequency (LF) power is an other frequency-domain parameter influenced by both sympathetic and parasympathetic activity and its interpretation remains complex. The LF/HF ratio is traditionally considered an index of sympathovagal balance. The stress index (SI) is a geometric measure, with high values indicating sympathetic activation and reduced variability. The two indices parasympathetic nervous system index (PNS index) and sympathetic nervous system index (SNS index) evaluate parasympathetic and sympathetic cardiac activity compared to normal values, and are obtained from other aforementioned aggregated metrics. The data were screened for noise segments, artifacts, or ectopic beats, and corrected when required using an automatic correction algorithm [10].

### Statistical analyses

Data analysis was done in R 4.0.4. HRV values are presented as average ± standard deviations. Statistical significance was evaluated with linear-mixed model using the subject unique ID as a random effect and the timepoint as a fixed effect using R package lmerTest (**p < 0.01).

## Results

Before the start of fasting, the group of 16 individuals (8 males and 8 females) had a mean age of 45 ± 11 years and a mean body mass index (BMI) of 26 ± 4 kg/m². After 11 days of fasting, participants experienced a significant reduction in body weight (75.9 ± 10.3 kg to 70.2 ± 9.4 kg; −5.7 ± 1.3 kg, *p* < 0.001). Systolic blood pressure decreased from 121.3 ± 15.7 mmHg to 114.4 ± 10.8 mmHg (−6.9 ± 13.4 mmHg, *p* = 0.04), and diastolic blood pressure did not significantly change, from 79.6 ± 8.8 mmHg to 76.1 ± 9.9 mmHg. Heart rate, as measured by electronic sphygmomanometer, increased slightly but not significantly from 67.2 ± 10.6 bpm to 69.2 ± 13.4 bpm (*p* = 0.52). Mental well-being improved significantly, with scores increasing from 53.4 ± 6.7 to 57.6 ± 8.5 (+4.2 ± 6.9, *p* = 0.029). Sleep quality, as measured by the Pittsburgh Sleep Quality Index, remained statistically unchanged (5.2 ± 2.0 to 4.9 ± 2.8; *p* = 0.700).

HRV recordings demonstrated high fidelity, with artifact rates consistently below 5%. We first aggregated HRV daily measurements into three broad periods (before, during, and after fasting) to reduce experimental noise (Table 1). Parasympathetic activity measured by RMSSD significantly increased from 27.16 ± 10.5 to 32.92 ± 17.65 ms after fasting compared to before (*p* = 0.01), suggesting enhanced vagal tone post-fasting. Sympathetic activity, reflected by the sympathetic nervous system (SNS) index, significantly decreased after fasting (*p* = 0.00007) indicating reduced sympathetic drive. Measures of overall heart rate variability, such as the standard deviation of NN intervals (SDNN) and the standard deviation along the line of identity of NN intervals (SD2), were not significantly modified by fasting. These findings suggest that fasting induces a pronounced shift toward parasympathetic dominance.

**Table 1:** HRV parameters reflecting ANS regulation during long-term fasting.

	Baseline (T0)	Fasting (T1)	Post-fasting (T2)	*p* values
HF power (ms2)	313.6 ± 269.7	337.7 ± 336.2	432.7 ± 443.3	T2–T0*; T2–T1*
LF power (ms2)	796.0 ± 727.3	723.3 ± 885.3	865.8 ± 1270.1	ns
LF/HF	3.83 ± 4.72	3.01 ± 2.76	2.74 ± 2.47	T2–T0*
MeanHR (bpm)	62.79 ± 7.36	62.86 ± 8.42	58.44 ± 7.68	T2–T0***; T2–T1***
MeanRR (ms)	968.8 ± 114.4	970.7 ± 123.4	1043.2 ± 128.6	T2–T0***; T2–T1***
PNSindex	-0.22 ± 0.68	-0.17 ± 0.86	0.31 ± 0.93	T2–T0***; T2–T1***
RESP (breaths/min)	13.95 ± 2.83	14.04 ± 2.25	13.59 ± 1.88	ns
RMSSD (ms)	27.16 ± 10.5	28.15 ± 17.43	32.92 ± 17.65	T2–T0*; T2–T1*
SD1 (ms)	19.26 ± 7.45	19.96 ± 12.4	23.35 ± 12.52	T2–T0*; T2–T1*
SD2 (ms)	44.08 ± 18.19	41.49 ± 24.75	43.99 ± 20.83	ns
SDNN (ms)	34.19 ± 13.41	32.73 ± 19.15	35.4 ± 16.72	ns
SI	12.9 ± 4.09	13.8 ± 5.37	12.43 ± 4.72	T2–T1*
SNSindex	0.39 ± 0.83	0.51 ± 1.16	0 ± 1.05	T2–T0**; T2–T1***

*p* values are for the statistical significance of group comparisons with repeated measures ANOVA followed by Tukey for multiple comparisons of means (*p < 0.05; **p < 0.01; ***p < 0.001). Values are average ± SD of aggregated HRV daily measurements into three broad periods (before, during, and after fasting).

*RMSSD (ms)* root mean square of successive differences between adjacent RR intervals, *SDNN (ms)* standard deviation of all normal-to-normal RR intervals, Low-frequency (LF) power (ms²), High-frequency (HF) power (ms²), LF/HF ratio, *MeanRR (ms)* average RR interval duration, *MeanHR (bpm)* average heart rate in beats per minute, *RESP (breaths/min)* respiratory rate; short-term variability 1 (SD1) and 2 (SD2), *PNSindex* parasympathetic nervous system index, *SNSindex* sympathetic nervous system index, *SI* stress index.

**Fig. 1:**
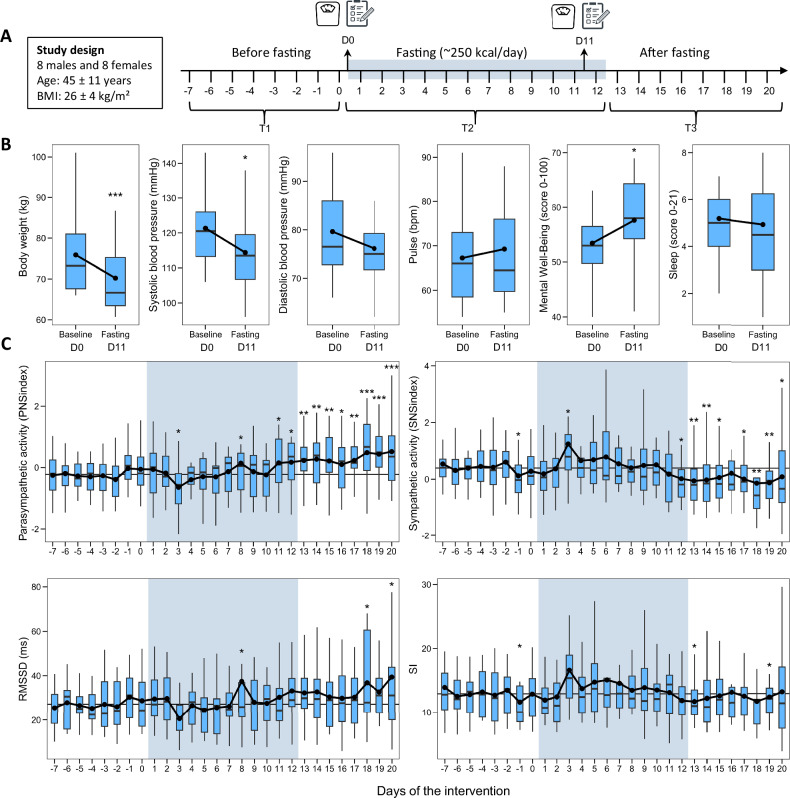
Effects of long-term fasting on clinical parameters of cardiovascular function and heart rate variability as a measure of autonomic nervous system tone. **A** Study design. **B** Changes in body weight (kg), systolic and diastolic blood pressure (mmHg), pulse (bpm), mental well-being (Warwick-Edinburgh Mental Wellbeing Scale), and sleep quality (Pittsburgh Sleep Quality Index) from baseline (D0) to the end of fasting (D11). Data are presented as boxplots showing the median and interquartile range. **C** The two indices PNSindex (parasympathetic nervous system index) and SNSindex (sympathetic nervous system index) evaluate parasympathetic and sympathetic cardiac activity, and the stress index (SI) cardiovascular stress. Statistical significance is from a linear-mixed model using the subject unique ID as a random effect and the timepoint as a fixed effect using R package lmerTest (*p < 0.05; **p < 0.01; ***p < 0.001). The blue area represents the fasting period, where day 1 is the first fasting day.

We also analysed these findings on a day-by-day basis to uncover the temporal pattern of autonomic shifts during the fasting period (Fig. 1). This detailed analysis highlights a nuanced response, with the early days of fasting marked by an increase in sympathetic cardiac activity. Additionally, the day-by-day evaluation confirms the sustained enhancement of parasympathetic control during the food reintroduction phase following fasting.

Mental well-being documented by the Warwick-Edinburgh Mental WellBeing Scale increased during the study from 53.4 ± 6.7 at baseline to 57.6 ± 8.5 at the end of the fasting period (*p* = 0.02). Quality of sleep as measured by the Pittsburgh Sleep Quality Index was statistically unchanged.

## Discussion

Our study, despite its small sample size, reveals for the first time the biphasic effects of long-term fasting on the autonomic nervous system. The early sympathetic activation observed during the first days of fasting aligns with prior studies and reflects an adaptive stress response aimed at mobilizing energy reserves [3].

The increase in RMSSD after fasting suggests that parasympathetic activity continued to strengthen during food reintroduction, possibly reflecting recovery from fasting-induced stress and enhanced autonomic flexibility. This enhancement of parasympathetic control can be connected to the beneficial effects of long-term fasting on mood enhancement and relaxation already documented [11]. The enhancement of parasympathetic dominance is known to heighten interoceptive sensitivity during fasting [12], which is the ability to detect and interpret physiological signals (e.g. heartbeats). Improved interoception could underlie psychological benefits commonly associated with fasting [13], including greater self-efficacy, mindfulness and self-compassion. These shifts in ANS regulation may have contributed to the observed improvement in mental well-being.

Further studies with larger, randomized cohorts are needed to confirm these results. Integrating additional biomarkers, such as inflammatory cytokines and neuroendocrine factors, could further elucidate the mechanisms underpinning these effects.

In conclusion, our results show that, in clinical settings, long-term fasting may hold promise for enhancing physiological resilience and autonomic regulation, particularly in stress-related disorders.

## Data Availability

The original contributions presented in the study are included in the article/Supplementary material; further inquiries can be directed to the corresponding author.
